# A Fault Diagnosis Method for 5G Cellular Networks Based on Knowledge and Data Fusion

**DOI:** 10.3390/s24020401

**Published:** 2024-01-09

**Authors:** Lingyu Zhao, Chuhong He, Xiaorong Zhu

**Affiliations:** College of Telecommunications and Information Engineering, Nanjing University of Posts and Telecommunications, Nanjing 210003, China; 2020010201@njupt.edu.cn (L.Z.); 1022010105@njupt.edu.cn (C.H.)

**Keywords:** 5G networks, graph convolutional neural network, generative adversarial network, naive Bayesian model, fault diagnosis

## Abstract

As 5G networks become more complex and heterogeneous, the difficulty of network operation and maintenance forces mobile operators to find new strategies to stay competitive. However, most existing network fault diagnosis methods rely on manual testing and time stacking, which suffer from long optimization cycles and high resource consumption. Therefore, we herein propose a knowledge- and data-fusion-based fault diagnosis algorithm for 5G cellular networks from the perspective of big data and artificial intelligence. The algorithm uses a generative adversarial network (GAN) to expand the data set collected from real network scenarios to balance the number of samples under different network fault categories. In the process of fault diagnosis, a naive Bayesian model (NBM) combined with domain expert knowledge is firstly used to pre-diagnose the expanded data set and generate a topological association graph between the data with solid engineering significance and interpretability. Then, as the pre-diagnostic prior knowledge, the topological association graph is fed into the graph convolutional neural network (GCN) model simultaneously with the training data set for model training. We use a data set collected by Minimization of Drive Tests under real network scenarios in Lu’an City, Anhui Province, in August 2019. The simulation results demonstrate that the algorithm outperforms other traditional models in fault detection and diagnosis tasks, achieving an accuracy of 90.56% and a macro F1 score of 88.41%.

## 1. Introduction

In recent years, mobile communication technology has rapidly evolved, and the scale of communication networks continues to expand. Nowadays, 5G networks have densely deployed nodes and a complex internal structure. In addition, Software-defined network (SDN) and network functions virtualization (NFV) technologies have been introduced to support network slicing to achieve new performances, such as elastic resource allocation and dynamic scheduling. Significantly, SDN constructs a centralized and controlled network by separating the control plane and forwarding plane. However, by using network-slicing technology to establish end-to-end logical networks and allocate network resources reasonably, 5G networks become more complex and challenging to maintain. It has been difficult to meet demand with traditional network operation and maintenance means. In particular, the blossoming business ecology in the 5G era has put higher requirements on the intelligence level of network operation and maintenance.

Network fault diagnosis is a common task undertaken by mobile communication operators, aiming to analyze the root causes of faults in communication networks. Before the widespread adoption of artificial intelligence and big data technologies, manual detection and diagnosis of network faults were the most commonly used methods by operators. In the early stages of research on network fault diagnosis, acquiring data sets of network faults was challenging and often required manually constructing relevant simulated network scenarios to complete the task of collecting network fault data. In [1], the authors focused on the study of self-healing functions in self-organizing networks (SONs) and proposed a fundamental cause analysis system based on a genetic fuzzy algorithm. Fuzzy logic can simulate the human thinking process by transforming input values into fuzzy sets easily understandable by humans through heuristic rules (fuzzy rules). The authors of [2] presented a design and evaluation method for a long-term evolution (LTE) network self-healing system, which is divided into three stages: establishing fault models and collecting labeled data, defining cause–symptom relationships, and designing a diagnostic system based on the first two aspects. In [3], P. Szilagyi et al. proposed a network fault detection and diagnosis framework involving less domain expert knowledge, which used key performance indicator (KPI) level as a measure of deviation from normal conditions and calculated the score for each fault by counting the frequency of abnormal KPIs, then made the final diagnosis decision based on the score. In [4], H. Mfula et al. studied an automatic network fault root cause analysis method using Bayesian theory, which did not need to be run manually and could combine domain expert knowledge for accurate and efficient automated fault diagnosis. The authors of [5] took into account the temporal dependencies between network metrics, explored the inter-dependencies between the network metrics of the primary serving cell and neighboring cells in the presence of network faults, and then compared them with stored historical faults to determine the root cause of faults. However, traditional network fault diagnosis methods require operations and maintenance personnel, along with relevant experts, to analyze and compare the collected network data with historical fault data in databases based on their work experience to determine the root causes of network faults. Nevertheless, when dealing with massive amounts of network data, these approaches are no longer practical and cannot achieve real-time network fault diagnosis. Additionally, relying solely on human knowledge for fault diagnosis may not be entirely accurate. In typical scenarios, operations and maintenance personnel may consider a specific KPI affected by network faults when it continuously exceeds a predefined threshold over a period or surpasses the threshold a certain number of times. However, in the context of modern communication networks with complex structures and large scales, this simple threshold judgment is evidently not precise, as the occurrence of network faults is no longer linearly mapped to individual KPIs. Finally, a fault diagnosis method completely reliant on expert knowledge and manual intervention inevitably leads to significant cost expenses. It is evident that traditional network fault diagnosis methods rely heavily on the accumulation of manual testing, experience, and skills, consuming substantial human and material resources, with lengthy optimization cycles that fall short of achieving the goal of cost reduction and efficiency improvement in network optimization tasks. Therefore, the adoption of intelligent methods, such as big data mining and machine learning in network fault diagnosis, emerges as a future trend.

Currently, academia has undertaken extensive research, employing various artificial intelligence and big-data-mining techniques to analyze network parameter data sets for efficient network fault diagnosis [6,7,8,9,10,11]. In [6], A. Gómez-Andrades et al. proposed an automatic LTE network diagnosis system based on unsupervised learning. It used self-organizing maps (SOMs) with Ward’s method to guarantee the quality of the solution through an iterative process. The authors in [7] introduced a data-driven methodology for fault detection and diagnosis (FDD) by integrating principal component analysis (PCA) with a Bayesian network (BN). In their approach, they employed correlation dimension (CD) to automatically select principal components (PCs) and utilized Kullback–Leibler divergence (KLD) and copula theory to develop a data-driven BN learning technique. The authors of [8] combined SoftMax neural networks and support vector machine (SVM), which could handle complex situations where multiple network faults exist simultaneously. In [9], the authors primarily addressed the issue of imbalanced data distribution in fault diagnosis. They present a novel imbalanced data classification method based on weakly supervised learning. The approach involves utilizing the bagging algorithm to randomly sample majority data, generating several relatively balanced subsets for training multiple SVM classifiers. Subsequently, these classifiers are employed to predict labels for unlabeled data, and samples predicted as belonging to the minority class are added to the original data set. An artificial intelligence network fault diagnosis system applied to LTE/5G wireless KPI management was proposed in [10], which used machine learning and deep learning to automatically detect wireless KPI statistics in specific cells with significant deviations in the probability density function for the standard KPI and alerted the network administrator to the possible causes of the related problems. In addition, Chen K. F. et al. delved into the examination of multi-fault scenarios and the diagnosis of fault severity levels within SONs in [11]. They employed a deep neural network featuring batch normalization to discern the various faults and their respective severity levels.

Meanwhile, as a branch of deep learning, methods based on graph convolutional network (GCN) have demonstrated outstanding performance in the field of fault diagnosis, particularly excelling in big data processing. These approaches have been preliminarily applied in the domain of mechanical fault diagnosis. In [12], GCN was used for fault diagnosis of power transformers by first forming association graphs between dissolved gas sample data using the k-nearest neighbor (KNN) method, and using the feature extraction capability of GCN to obtain complex and complicated mapping relationships between dissolved gas and fault types. Moreover, as one of the key technologies for achieving cognitive intelligence, knowledge graphs (KGs) have gradually begun to be applied in the field of fault diagnosis in recent years. The authors of [13] established a knowledge-based question-and-answer system for fault diagnosis of the Electric Information Collection System based on knowledge graph technology to meet the requirements of efficient and intelligent decision making under massive operation and maintenance data. In [14], the authors summarized the latest developments in knowledge-based fault diagnosis in industrial Internet of things (IIoTs) systems through building knowledge bases with ontologies and applying deductive/inductive reasoning. They also discussed unresolved issues regarding future decentralized implementations of fault diagnosis. Considering the respective advantages of methods based on GCN and knowledge-based methods, the authors of [15] integrated the prior knowledge of the system of interest with GCN for fault diagnosis. They first employ the structural analysis (SA) method to pre-diagnose the fault and then transform the pre-diagnosis results into an association graph. Subsequently, the graph and measurements are fed into the GCN model for training.

Compared to traditional fault diagnosis methods, solutions integrating cutting-edge technologies such as big data mining and machine learning into the fault diagnosis process have significantly optimized the efficiency and performance of network fault diagnosis. However, most current fault diagnosis methods based on knowledge, big data, or machine learning still have some drawbacks. Firstly, data-driven methods often rely on large-scale labeled data sets, and obtaining substantial labeled data in the field of network fault diagnosis can be challenging, resulting in the underutilization of unlabeled data and the waste of potential information. Secondly, relying solely on machine learning methods, especially in the absence of domain-specific knowledge, may limit the model’s generalization capability, thereby affecting the diagnostic effectiveness for new types of faults or complex scenarios. Finally, the data in 5G communication networks is both vast and complex, necessitating significant amounts of data and time for accurate fault diagnosis using machine learning methods. The process of collecting data through drive test (DT) techniques in the existing network and manually labeling categories can also be expensive and time consuming. It is worth noting that as the uncertainty and complexity of mobile communication networks increase, these solutions cannot be seamlessly transferred to the current network environment. Therefore, it is significant to integrate knowledge-based and data-based methods, leveraging their respective strengths while mitigating their individual shortcomings, to enhance the efficiency and reliability of 5G communication network fault diagnosis.

To address these issues, we propose a knowledge- and data-fusion-based fault diagnosis method for 5G cellular networks. This method aims to accurately and quickly identify possible network fault types and accelerate the recovery of network faults. We use GAN to expand the real network data set and then use NBM combined with expert knowledge to pre-diagnose the data set. Subsequently, a topological association graph is generated based on the pre-diagnosis results, while improvements are made to the GCN model. This enhancement enables the GCN to control both the pre-diagnosis results and the size of the training data set during model training, allowing for an assessment of their respective impacts on training accuracy. The main contributions of this study are as follows.

- To address the problems of sparse labeled samples and uneven distribution of sample classes in the actual collected network parameter data set, we proposed a method based on the generative adversarial network to expand the data set. It not only helps balance the data set but also ensures that the subsequent model training aligns with the demands of real-world dynamic network scenarios.
- In order to improve the accuracy of constructing the adjacency matrix using a single GCN method, this paper introduces the naive Bayes method and expert knowledge to construct a topological correlation graph. The traditional topological correlation graph generated based on the Euclidean distance between individual nodes is not entirely accurate. Therefore, we use the pre-diagnosis result set to enhance the accuracy of the topological correlation graph and make the model more interpretable.
- A GCN-based fault diagnosis model is constructed by inputting the generated topological association graph and the expanded training data set into the GCN model for model training. The GCN realizes information aggregation between nodes and their neighboring nodes by using the graph convolution layer with strong learning capability to obtain new feature representations of nodes and learn the complex non-linear relationships between network KPI parameters and fault types based on these higher-order features. Meanwhile, the GCN model can adjust the impact of pre-diagnostic prior knowledge and training data set size on the accuracy of the GCN model during training.

The remainder of this paper is organized as follows. In Section 2, we provide a detailed description of the considered network scenario and the network parameter data set. In Section 3, based on the overall framework of the proposed algorithm, we first introduce how to preprocess the original data set in Section 3.1. Subsequently, in Section 3.2, we propose a GAN-based approach to expand the original data set and balance the number of samples for different fault types. Following that, in Section 3.3, we suggest using NBM combined with expert knowledge to pre-diagnose the network fault data set and generate the corresponding topological association graph between the data. Next, in Section 3.4, we construct a GCN-based fault diagnosis model. In Section 4, we present the simulation results of our proposed algorithm and compare its accuracy with other algorithms. Lastly, in Section 5, we conclude this paper.

## 2. System Model

**Figure 1:**
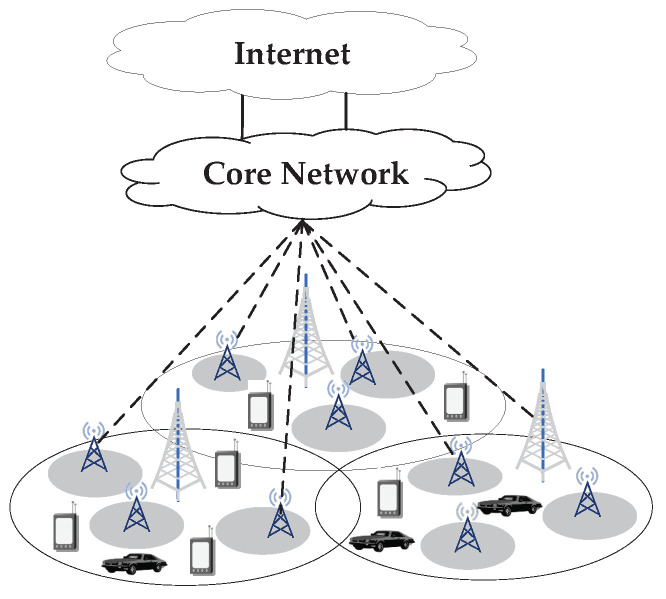
Dense heterogeneous cellular network.

In this paper, we consider the network application scenario of a dense heterogeneous 5G cellular network consisting of one high-power macro base station and many low-power micro base stations, as shown in Figure 1, in which the proposed algorithm is used to accurately detect faults and find out the root cause of network faults, to prevent the continuous negative impact on network operation caused by network faults.

To avoid the over-idealization of model diagnosis results caused by using a simulation data set to train the model, we use the data set collected by minimization of drive tests (MDT) technology under real network scenarios, which is the real user-side data collected by the company concerned in Lu’an City, Anhui Province, in August 2019. According to the network optimization rules of the company’s network optimization staff and the relevant definitions in the problem point list, the network faults in the data set are divided into a total of eight categories, namely signal leakage of the indoor distribution system, measurement threshold abnormality, large station spacing, mode-3 interference, handover threshold abnormality, pilot pollution, overlapping coverage, and missing neighbor.

Since the data set considers the influence of neighboring base stations on the current base station, the data set also contains the values of relevant KPI parameters of neighboring base stations of the main service base station recorded by the measurement terminals. After removing the geographic location parameters such as latitude (LAT) and longitude (LNG) of the measurement terminals and the network-optimization-independent parameters such as mobile network code (MNC), 14 KPIs are retained, which are RSSI, RSSI0, RSSI1, RSRP, RSRP0, RSRP1, RSRQ, RSRQ0, RSRQ1, SINR, SINR0, SINR1, RSRQ_1, and RSRP_1. Taking RSRP as an example, RSRP, RSRP0, and RSRP1 indicate the value of the primary service base station received at the current measurement terminal, channel 0 and channel 1 in the terminal, respectively. RSRP_1 indicates the RSRP value of the largest neighboring base station received by the measurement terminal. Table 1 shows the explanations of relevant KPI parameters.

## 3. Knowledge- and Data-Fusion-Based 5G Network Fault Diagnosis Algorithm

**Figure 2:**
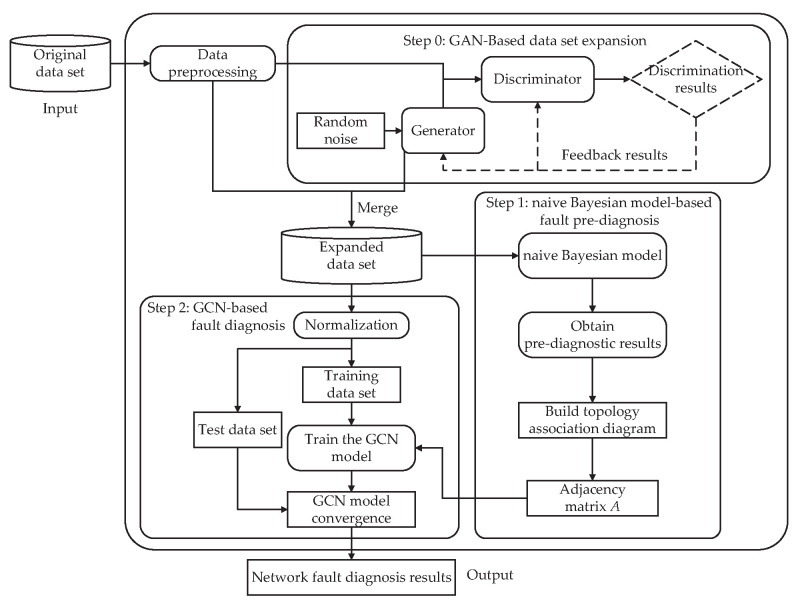
Fault diagnosis algorithm flow diagram.

The overall flow of the proposed algorithm’s operation is shown in Figure 2. The actual network parameter data set collected with few labeled samples is firstly preprocessed. The data are input into GAN in turn according to the fault category to obtain a large amount of labeled simulated data under different network fault categories, and the actual and simulated data sets are merged to obtain the expanded data set. Next, we perform fault diagnosis. The fault diagnosis process is divided into two stages: In the first stage, the data set is pre-diagnosed with a classification task using NBM, and then the adjacency matrix A of the data set is obtained based on the results of the pre-diagnosis. In the second stage, the trained GCN network model is obtained by using the adjacency matrix A combined with the GCN model for training. According to the GCN model, the final network fault diagnosis is performed on the data set, and the diagnosis results of the network fault are output.

### 3.1. Data Preprocessing

**Figure 3:**
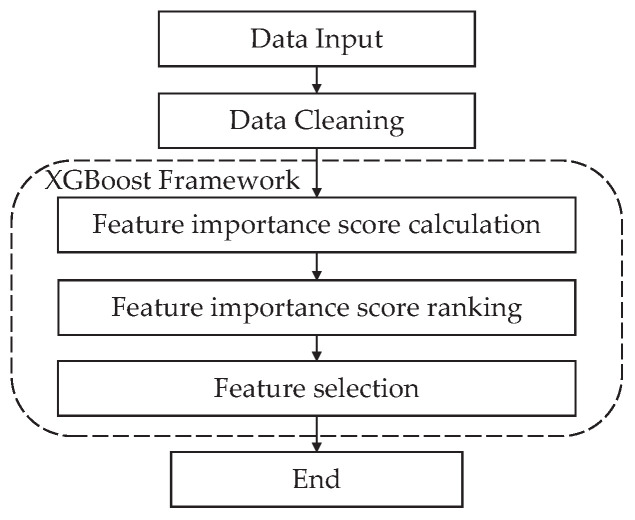
Flow diagram of data preprocessing.

Some data samples in the actual data set have duplicate or missing problems, so these useless data need to be removed from the data set. XGBoost [16] integrates the prediction results of many single-tree models to improve its performance and then evaluates the importance of the feature parameters according to the splitting times of feature attributes in each tree, making the feature selection results more reasonable. Therefore, we choose XGBoost to address the optimal combination of feature parameters. As shown in Figure 3, by constructing multiple decision tree models, we obtain corresponding importance scores for each feature parameter and subsequently rank all feature parameters in descending order. Ultimately, different numbers of feature combinations are selected based on the ranking results to achieve the optimal feature combination selection for the data set.

Assuming the original data set has a total of *d* feature parameters, after employing XGBoost for feature selection, the number of feature parameters becomes $d_{0}\left(0<d_{0}<d\right)$. Simultaneously, normalization is performed on the data set after feature selection before training the GCN model, mapping the values of each feature parameter to the [0, 1] interval. This process helps prevent higher values of feature parameters from dominating the entire model training process. In this paper, the maximum-minimum normalization is performed for each feature parameter as

(1) $${\tilde{x}}_{i}=\frac{x_{i}-\min\left(x_{i}\right)}{\max\left(x_{i}\right)-\min\left(x_{i}\right)},i=1,2,\ldots ,z_{0},$$

where $x_{i}$ and ${\tilde{x}}_{i}$ denote the *i*-th feature before and after normalization, and $\max(x_{i})$ and $\min(x_{i})$ denote the maximum and minimum values of the *i*-th feature attribute, respectively.

We define the preprocessed network fault data set as $\left\{\left(x_{1},y_{1}\right),\left(x_{2},y_{2}\right),\ldots ,\left(x_{n},y_{n}\right)\right\}$, where $x_{i}=\left[{\mathrm{KPI}}_{i,1},{\mathrm{KPI}}_{i,2},\ldots ,{\mathrm{KPI}}_{i,d_{0}}\right]\in R^{d_{0}}$ denotes a vector of characteristic parameters reflecting the network condition in the current environment through $d_{0}$ KPIs with corresponding network fault category label $y_{i}\in Y$. $Y=\{y_{1},y_{2},\cdots ,y_{L}\}$ is the set of network fault categories, where L=8 according to the previous section, representing eight different network fault categories defined in the data set.

### 3.2. GAN-Based Sample Expansion and Balance

The original data set has limitations, including limited sample size, uneven distribution of data samples, and scarcity of labeled data for certain categories. Therefore, this paper utilizes GAN-based methods to enhance real data sets. GAN was first proposed as a generative model by Ian Goodfellow [17]. It has attracted much attention upon its introduction and has been shown to perform well in expanding data sets to improve model classification accuracy [18]. The GAN model requires only a certain number of actual data samples. By reasonably training the GAN model, simulated network parameter data matching the real network failure scenarios can be generated. Moreover, after using the GAN model to expand the data under each network failure category, we try to make the number of samples under each category as balanced as possible.

**Figure 4:**
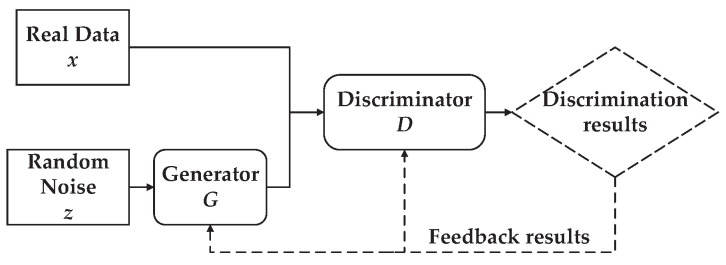
The GAN framework.

As shown in Figure 4, GAN is essentially an adversarial process generated by two neural network models competing with each other. The generator *G* generates fake simulated data after inputting raw random noise obeying a specific distribution. The discriminator *D* tries to perform a binary classification task to distinguish actual data from the fake data generated by the generator. There is no fixed choice of neural network models for the generator and discriminator, and two multilayer perceptrons are chosen in [17] to complete the training of the GAN by updating the network parameters. When training the GAN, the discriminator *D* is generally trained first, and the generator *G* is trained alternatively. According to [17], the objective optimization function of the GAN can be expressed as

(2) $$\begin{array}{cc} \min_{G}\max_{D}V\left(D,G\right) & =E_{x∼P_{d}\left(x\right)}\left[\log D\left(x\right)\right]+E_{z∼P_{z}\left(z\right)}\left[\log\left(1-D\left(G\left(z\right)\right)\right)\right], \end{array}$$

where $P_{d}\left(x\right)$ is the probability distribution of the real data *x*, which in this paper is denoted as the probability distribution of the KPI parameters in the actual network scenarios. $P_{z}\left(z\right)$ is the probability distribution of the random noise *z* input to the generator.

It can be observed that the objective function of GAN is a minimax optimization problem, essentially composed of the superposition of the loss functions of the generator and discriminator. Specifically, the loss functions for the generator *G* and the discriminator *D* are defined as follows:

(3) $$\begin{array}{cc} L_{G} & =E_{z∼P_{z}\left(z\right)}\left[\log\left(1-D\left(G\left(z\right)\right)\right)\right],\\ L_{D} & =-(E_{x∼P_{d}\left(x\right)}\left[\log D\left(x\right)\right]+E_{z∼P_{z}\left(z\right)}\left[\log\left(1-D\left(G\left(z\right)\right)\right)\right]), \end{array}$$

On the one hand, for the generator *G*, if the current data are virtual data generated by the generator based on *z*, the generator aims for the discriminator’s output probability D(G(z)) to tend towards a positive judgment of 1, thereby deceiving the discriminator and minimizing log(1−D(G(z))).

On the other hand, for the discriminator *D*, if the current data are real, the discriminator expects to provide a positive judgment close to 1, maximizing log(D(x)). If the current data are virtual data generated by the generator, the discriminator expects to give a negative judgment close to 0 for fake data, maximizing log(1−D(G(z))).

However, GAN also has some problems. In the actual training process of GAN, assuming that the discriminator has first reached the approximate optimal state, GAN will introduce Jensen–Shannon divergence, a distance metric, to rewrite the loss function of the generator, and the optimization goal of the generator is equivalent to minimizing the Jensen–Shannon divergence between the distribution of actual data and generated data. Since the generation level of the generator is poor in this case, it is challenging to generate a non-negligible overlap between the distribution of generated simulated data and the actual data, and the Jensen–Shannon divergence is equal to a constant. Moreover, the generator will experience gradient disappearance in the process of optimization. It cannot be further trained without getting gradient information updates, which eventually leads to the difficulty of GAN convergence.

To solve this problem, WGAN proposed replacing the Jensen–Shannon divergence in the original optimization objective by minimizing the Wasserstein distance between the generated and actual samples [19]. The Wasserstein distance is smoother and provides continuous and effective gradients during the training process, thus fundamentally solving the problem of GAN gradient disappearance. Since it is difficult to compute the lower bound when solving the Wasserstein distance in practice, WGAN indirectly satisfies the 1-Lipschitz restriction by ensuring that the parameters of the discriminator network are bounded during the training process, thus achieving the goal of simplifying the computation of the Wasserstein distance. Finally, the discriminator is re-modeled as a neural network used to fit the Wasserstein distance between the generated data and the actual data distribution.

Since WGAN limits the range of values of the discriminator network parameters in updating the parameters of the neural network model, it will make the neural network unable to learn complex function expressions and significantly reduce the performance capability of the discriminator. Therefore, WGAN-GP proposes to avoid the weight restriction on the discriminator network by adding a gradient penalty term to the original WGAN discriminator loss function (the generator loss function is not modified) [20], while ensuring that the 1-Lipschitz restriction is satisfied. Specifically, according to [20], the discriminator loss function in WGAN-GP is improved as follows:

(4) $$\begin{array}{cc} L_{D} & =E_{\tilde{x}∼P_{g}}\left[D\left(\tilde{x}\right)\right]-E_{x∼P_{d}}\left[D\left(x\right)\right]+\rho E_{\hat{x}∼P_{\hat{x}}}\left[{\left({∥\nabla_{\hat{x}}D\left(\hat{x}\right)∥}_{2}-1\right)}^{2}\right], \end{array}$$

where $P_{g}$ is the distribution of the generator-generated data, ρ∈[0,+∞) is the penalty term coefficient, and we take the default value of 10 referring to [20]. $P_{\hat{x}}$ is the distribution of the sampled data in the penalty term, and the sample $\hat{x}$ is obtained by linear interpolation sampling between the real sample *x* and the generated sample $\tilde{x}$, thus avoiding traversing the whole sample space for sampling. $E_{\hat{x}∼P_{\hat{x}}}\left[{\left({∥\nabla_{\hat{x}}D\left(\hat{x}\right)∥}_{2}-1\right)}^{2}\right]$ is the penalty term, which will force the discriminator’s gradient ${∥\nabla_{\hat{x}}D\left(\hat{x}\right)∥}_{2}$ at the sample point $\hat{x}$ to be as close to 1 as possible during the training process of WGAN-GP so that the discriminator network satisfies the 1-Lipschitz constraint.

The implementation of WGAN-GP is described in Algorithm 1, where the number of discriminator training iterations with the fixed generator is $n_{\mathrm{critic}}$, and the size of the batch is *m*. The network parameters of generator and discriminator are optimized in WGAN-GP by using Adam’s algorithm, where the hyperparameters of Adam’s algorithm are defined as follows: α is the learning rate, $\beta_{1}$ is the exponential decay rate of first-order moment estimation, and $\beta_{2}$ is the exponential decay rate of second-order moment estimation. In this paper, we specify $n_{\mathrm{critic}}=100$, α=0.001, $\beta_{1}=0.9$, and $\beta_{2}=0.999$.
**Algorithm 1** WGAN-GP.1:Initialize: discriminator parameter $w_{0}$, generator parameter $\theta_{0}$.2:**while** generator parameter θ has not converged **do**3:   **for** $t=0,\ldots ,n_{\mathrm{critic}}$ **do**4:     **for** i=1,…,m **do**5:        Sampling real data $x∼P_{r}$, latent variable z∼p(z), a random number ε∼U[0,1]6:        $\tilde{x}\leftarrow G_{\theta }\left(z\right)$ //Generated data for generator7:        $\hat{x}\leftarrow \varepsilon x+\left(1-\varepsilon \right)\tilde{x}$ //Sampling data in penalty term8:        $L^{\left(i\right)}↔D_{w}(\tilde{x}-D_{w}\left(x\right)+\rho {\left({∥\nabla_{\hat{x}}D_{w}\left(\hat{x}\right)∥}_{2}-1\right)}^{2}$ //Calculate the discriminator           //loss function9:       **end for**10:     $w\leftarrow \mathrm{Adam}(\nabla_{w}\frac{1}{m}\begin{array}{c} \sum_{i=1}^{m}L^{\left(i\right)} \end{array},w,\alpha ,\beta_{1},\beta_{2})$//Update discriminator parameter11:   **end for**12:   Sample a batch of latent variables ${\left\{z^{\left(i\right)}\right\}}_{i=1}^{m}∼p_{(}z)$13:   $\theta \leftarrow \mathrm{Adam}(\nabla_{w}\frac{1}{m}\begin{array}{c} \sum_{i=1}^{m}-D_{w}\left(G_{\theta }\left(z\right)\right) \end{array},\theta ,\alpha ,\beta_{1},\beta_{2})$//Update Generator parameter14:**end while**

### 3.3. Naive-Bayesian-Model-Based Fault Pre-Diagnosis

#### 3.3.1. Naive Bayesian Model

A Bayesian Network (BN) [21] is an acyclic directed graph with the advantage of using probabilistic statistics to classify sample data, thus effectively modeling the uncertainty inherent in human reasoning. Moreover, as a probabilistic model, BN is suitable for handling extensive data sets with complex probabilistic combinations, such as the network parameter data set. According to [21], BN can be represented as

(5) $$\mathrm{BN}=(G_{B},P),$$

where $G_{B}$ denotes an acyclic directed graph, the nodes in the chart are usually represented by a set of attribute variables $X=\{X_{1},X_{2},\ldots ,X_{n}\}$, and the edges in the graph represent the dependencies between these attributes. The network parameter *P* consists of the probability distributions of all nodes in the chart, representing the dependent probability of each node under the influence of its parent node. Each node corresponds to a conditional probability table, which can be expressed as $P(X_{i}|\pi \left(X_{i}\right))$, where $\pi (X_{i})$ denotes the set of parents of the attribute variable $X_{i}$. Thus, the set P defines a unique joint probability distribution over *X*, which is denoted as

(6) $$P\left(X\right)=P\left(X_{1},\ldots ,X_{n}\right)=\prod_{i=1}^{n}P\left(X_{i}|\pi \left(X_{i}\right)\right).$$

Once the structure $G_{B}$ and parameter *P* of the BN are determined, the construction of the BN is completed. Then, the joint probability of the BN can be used for subsequent posterior probability inference to complete tasks such as attribute value prediction and category classification.

The BN structure used in this paper is naive Bayes [22]. The reason why naive Bayes is chosen as the algorithm used in the first stage of the pre-diagnosis process is that the relationship between multiple possible causes that lead to network faults in cellular networks is uncertain. Therefore, these causes of network problems can be expressed in terms of probabilities, and naive Bayes is suitable for dealing with this kind of situation.

The NBM consists of a single parent node *Y* and *M* child nodes $X=\{X_{1},X_{2},\ldots ,X_{d_{0}}\}$. In the network fault diagnosis scenario of this paper, the parent node can be modeled as the network faults present in the network $Y=\{y_{1},y_{2},\ldots ,y_{L}\}$. At the same time, the child nodes $X_{1},X_{2},\ldots ,X_{d_{0}}$ represent each KPI feature parameter variable in the data set after feature selection, respectively. According to [22], naive Bayes is a probabilistic model based on Bayes’ theorem, which can be expressed as

(7) $$P\left(y_{i}|X\right)=\frac{P\left(y_{i}\right)P\left(X|y_{i}\right)}{P(X)},$$

where $P(y_{i}|X)$ is the posterior probability, which represents the probability of occurrence of $y_{i}$ given the observed *X*. $P(X|y_{i})$ is the likelihood probability, which represents the probability of occurrence of *X* given the already observed $y_{i}$. $P(y_{i})$ represents the prior probability. P(X) is the full probability formula concerning *X*.

Since P(X) is constant in the calculation of all parts of Equation (7), it can be ignored. In addition, since the naive Bayes makes a strong assumption of conditional independence on the conditional probability distribution, Equation (7) can be further expressed as

(8) $$P\left(y_{i}|X\right)\propto P\left(y_{i}\right)\prod_{j=1}^{M}P\left(x_{j}|y_{i}\right),$$

where $x_{j}$ denotes the specific value taken in *X* under the *j*-th feature parameter. From the perspective of network fault diagnosis, given the vector of input feature parameters representing the network state, for the defined set of fault causes $Y=\{y_{1},y_{2},\ldots ,y_{L}\}$, each posterior probability distribution $P(y_{i}|X)$ is calculated using the NBM, and the network fault category that makes the maximum posterior probability is selected as the network fault $h^{*}\left(X\right)$ suffered by the current network. Therefore, $h^{*}\left(X\right)$ is defined as

(9) $$h^{*}\left(X\right)=\arg\max_{y_{i}\in Y}P\left(y_{i}\right)\prod_{j=1}^{M}P\left(x_{j}|y_{i}\right).$$

In reality, solving Equation (9) will involve multiplying multiple conditional probabilities, which are usually smaller probability values. Therefore, to avoid underflow errors, we convert Equation (9) into the logarithmic form:

(10) $$h^{*}\left(X\right)=\arg\max_{y_{i}\in Y}\left[\log P\left(y_{i}\right)+\sum_{j=1}^{M}\log P\left(x_{j}|y_{i}\right)\right].$$

The two items $P(x_{j}|y_{i})$ and $P(y_{i})$ denote the evidence used in the process of naive Bayesian inference. To avoid bias in the calculation of probabilities and the situation where the probability value is equal to zero, we estimate the prior probability $P(y_{i})$ and the conditional probability $P(x_{j}|y_{i})$ by Laplacian smoothing:

(11) $$\begin{array}{c} P\left(y_{i}\right)=\frac{D_{y_{i}}+1}{D_{t}+L}, \end{array}$$

(12) $$\begin{array}{c} P\left(x_{j}|y_{i}\right)=\frac{D_{y_{i},x_{j}}+1}{D_{y_{i}}+S_{j}}. \end{array}$$

where $D_{t}$ is the total number of samples contained in the training data set; $D_{y_{i}}$ is the total number of samples in the training set that are in the $y_{i}$ case of network failure; $D_{y_{i},x_{j}}$ denotes the total number of samples in the training set that are in the $y_{i}$ case of network failure, and the *j*-th KPI parameter takes the value $x_{j}$; *L* is the total number of previously defined network failure categories; and $S_{j}$ is the total number of all possible values of the *j*-th KPI number of values, assuming that in our data set, a specific discrete KPI attribute parameter can be measured by three discrete values of high, medium, and low. In this case, $S_{j}=3$.

It is worth pointing out that the main difficulty in the process of fault pre-diagnosis based on naive Bayes is that the KPIs in the data set used in this paper are all continuous-type variables, so the conditional probability density function of the continuous-type KPIs needs to be known when calculating the conditional probability $P(x_{j}|y_{i})$. However, this is often difficult to obtain in reality. In addition, considering the sample data are relatively small, using discrete KPI fetches would make the diagnosis of the naive Bayes more accurate than using the continuous probability density function of the KPI, which is also more reasonable for the actual network parameter data set [23]. Therefore, we need to choose a discretization method to determine the threshold value of the KPI first, and then map the continuous KPI to a discrete interval to achieve the discretization of the KPI. In this paper, we use the expert’s empirical knowledge for the task of KPI discretization, and the specific discretization rules will be given and explained in Section 4.

Finally, according to the discretized KPI attributes, the naive Bayes classifier is trained by reasonably dividing the training data set, and the trained NBM is used to classify the remaining data to obtain the total pre-diagnosis result label set $\hat{C}=\left\{{\hat{c}}_{1},{\hat{c}}_{2},\ldots ,{\hat{c}}_{N}\right\}$ in the first stage, where *N* denotes the total number of samples in the data set after the WGAN-GP expansion.

#### 3.3.2. Topological Association Diagram Construction

After obtaining the pre-diagnostic result set $\hat{C}$, we construct the topological association graph. The topological association graph is mathematically represented by the adjacency matrix A, which can intuitively reflect the connection relationship between nodes in the diagram and plays an essential role in the subsequent training process of the GCN model.

It is noteworthy that since the pre-diagnosis results derived from the naive Bayes are not entirely accurate, $\hat{C}$ will not be directly used as the final diagnosis result in the next part of this paper, nor will the data with the labeling information derived from the pre-diagnosis be used as the labeling training data for the subsequent GCN. The pre-diagnosis results are only used for the construction of A.

Now, let us obtain A of the sample node data in the data set based on $\hat{C}$. In this paper, it is specified that in $\hat{C}$, data diagnosed as having the same network fault type are connected in the graph, while data with different network fault types are not connected, which means the element in A can be expressed as

(13) $$A_{i,j}=\left\{\begin{array}{c} \begin{array}{cc} 1, & \;\mathrm{if}\;{\hat{c}}_{i}={\hat{c}}_{j}\;\mathrm{and}\;i\neq j\\ 0, & \;\mathrm{otherwise}. \end{array} \end{array}\right.$$

According to Equation (13), the topological association graph is a graph composed of *L* mutually independent subgraphs, and *L* is the number of previously predefined network fault types.

### 3.4. GCN-Based Fault Diagnosis Model

We will focus on constructing a GCN-based network fault diagnosis model. First, as shown in Equation (14), the feature matrix $X\in R^{n\times d_{0}}$ can be constructed based on the data set obtained after data preprocessing in Section 2, where *n* denotes the number of data samples and assumes that the first *l* data $x_{i}\left(1\leq i\leq l\right)$ in the data set are labeled data with category label $y_{i}$, while the remaining data $x_{i}\left(l+1\leq i\leq n\right)$ are unlabeled data with category label $y_{i}=0$.

(14) $$X={\left(\begin{array}{ccc} {\mathrm{KPI}}_{1,1} & {\mathrm{KPI}}_{1,2}\cdots & {\mathrm{KPI}}_{1,d_{0}}\\ \vdots & \ddots & \vdots \\ {\mathrm{KPI}}_{l,1} & {\mathrm{KPI}}_{l,2}\cdots & {\mathrm{KPI}}_{l,d_{0}}\\ {\mathrm{KPI}}_{l+1,1} & {\mathrm{KPI}}_{l+1,2}\cdots & {\mathrm{KPI}}_{l+1,d_{0}}\\ \vdots & \ddots & \vdots \\ {\mathrm{KPI}}_{n,1} & {\mathrm{KPI}}_{n,2}\cdots & {\mathrm{KPI}}_{n,d_{0}} \end{array}\right)}_{n\times d_{0}}.$$

Next, the feature matrix X and the adjacency matrix A obtained in the previous section are used as inputs to the GCN:

(15) input=(X,A).

In addition, according to the encoding rules as shown in Table 2, we encode the category labels of all labeled data in the data set, while the category labels of all unlabeled data are represented as zero vectors. Based on this, the label vectors of all data are combined into a label matrix $Y\in R^{n\times c}$, which is used for subsequent computation of the cross-entropy loss function. Here, *c* is the predefined number of network failure categories, and in this paper, c=8, as described in Section 2.

In the GCN, the forward excitation propagation formula defined in the single-layer graph convolution layer is:

(16) $$H^{\left(l+1\right)}=\sigma \left({\tilde{D}}^{-\frac{1}{2}}\tilde{A}{\tilde{D}}^{-\frac{1}{2}}H^{\left(l\right)}W^{\left(l\right)}\right),$$

where σ is the activation function, $\tilde{D}$ is the degree matrix of matrix $\tilde{A}$, and each element on its main diagonal is obtained by summing all elements of the corresponding row in matrix $\tilde{A}$, while all elements outside the main diagonal are zero. $W^{\left(l\right)}$ is the trainable weight matrix in layer *l*, which is essentially the convolutional kernel filter parameter matrix. The parameters in $W^{\left(l\right)}$ can be updated during the training process of GCN by error back-propagation and according to the gradient descent method. $H^{\left(l\right)}$ is the input node feature matrix of the *l*-th layer graph convolution layer. For the input layer, $H^{\left(0\right)}$ is equal to the initial node feature matrix X. In addition, the matrix $\tilde{A}$ is defined as

(17) $$\tilde{A}=A+\lambda I_{n},$$

where A is the adjacency matrix, $I_{n}$ is the unit matrix, and λ is the weight coefficient that is positively correlated with the size of the training set, which is specifically defined in this paper as $\lambda =1+re^{r}$, where *r* denotes the proportion of the labeled training set to the size of the total data set.

**Figure 5:**
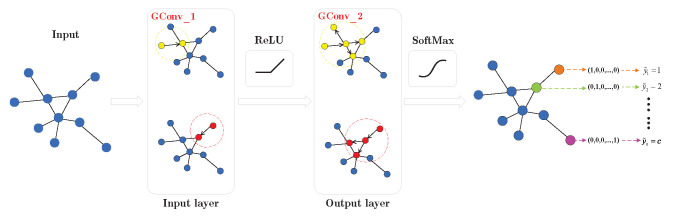
Graph convolutional neural network model.

Finally, we obtain the output matrix $Z\in R_{r}^{n\times c}$ of the graph convolutional neural network. Additionally, to comprehensively illustrate the network structure and processing procedure of the GCN in this paper, Figure 5 presents a GCN model consisting of two graph convolutional layers. For ease of explanation, in the actual GCN model, we refer to the 0-th graph convolutional layer as the first graph convolutional layer and so forth.

As shown in Figure 5, we first calculate $\hat{A}={\tilde{D}}^{-\frac{1}{2}}\tilde{A}{\tilde{D}}^{-\frac{1}{2}}$ in Equation (16), where $\hat{A}$ represents the normalized symmetric adjacency matrix, addressing numerical instability in the convolution operation. Analysis reveals that the matrix $\hat{A}$ contains association information for each node and its neighboring nodes. Therefore, $\hat{A}X$ is utilized to aggregate the feature attributes of each node and its neighbors. Subsequently, by multiplying it with the trainable weight matrix $W^{\left(0\right)}$, a new set of node features, $\hat{A}{\mathrm{XW}}^{\left(0\right)}$, is obtained. Finally, an activation function is selected for the new feature matrix to get the output feature matrix $H^{\left(1\right)}$ of the first graph convolution layer. The new node feature representation learned by the first graph convolution layer is:

(18) $$H^{\left(1\right)}=\mathrm{ReLU}\left(\hat{A}{\mathrm{XW}}^{\left(0\right)}\right).$$

Stacking multiple graph convolution layers allows the aggregation of feature attribute information from neighboring nodes in higher-order neighborhoods. Therefore, we use the output $H^{\left(1\right)}$ of the previous graph convolution layer as the input for the next graph convolution layer. After the second graph convolution layer, another set of node features, $\hat{A}H^{\left(1\right)}W^{\left(1\right)}$, is learned. It is noteworthy that the GCN constructed in Figure 5 uses only two graph convolution layers. Thus, the output feature matrix of the second graph convolution layer should have the same size as the label matrix Y, indicating a change in the feature vector dimension of nodes in the graph. Finally, the feature matrix is processed through the SoftMax activation function to obtain the ultimate output:

(19) $$Z=\mathrm{SoftMax}(\hat{A}H^{\left(1\right)}W^{\left(1\right)}),$$

where $W^{\left(1\right)}$ is the weight matrix of the second graph convolution layer. The SoftMax activation function needs to be applied to each row of the feature matrix $\hat{A}H^{\left(1\right)}W^{\left(1\right)}$.

Since we consider the network fault diagnosis task as a node classification task using GCN, we finally need to output a category label for each node. Therefore, the structural design of the network does not need to use the full connected layers like the traditional CNN but only needs to set the activation function on the last layer of the graph convolution layer as a SoftMax function.

The output result matrix $Z=[Z_{1},Z_{2},\ldots ,Z_{n}]$, and its representation is similar to the label matrix Y. Each row vector $Z_{i}\left(1\leq i\leq n\right)$ in Z corresponds to the predicted final network failure class of the sample node $x_{i}$ in the original data set. Specifically, for $Z_{i}=\left[Z_{i,1},Z_{i,2},\ldots ,Z_{i,c}\right]$, the predicted label of the sample node $x_{i}$ is ${\tilde{y}}_{i}=\arg\max_{1\leq j\leq c}Z_{i,j}$.

In the GCN training process, it is finally necessary to calculate the cross-entropy loss function from the labeled samples in the training set and perform the error backward propagation to optimize the weights of the weight matrix in each graph convolution layer according to the gradient descent method. The cross-entropy loss function can be expressed as

(20) $$L=-\sum_{i=1}^{l}\sum_{j=1}^{c}Y_{i,j}\ln Z_{i,j},$$

where *l* is the number of samples with labels, *c* is the total number of network fault categories defined before, and Y is the label matrix of nodes defined before.

This paper uses accuracy and macro F1 score as two commonly used evaluation metrics. Macro F1 score is a performance indicator that combines accuracy and recall. Accuracy represents the accuracy between the predicted value and the label value. Recall is the calculation of the proportion of correctly predicted categories, subsequently taking the average of the proportions of all categories. The calculation formula is as follows:

(21) $$\begin{array}{cc} Accuracy & =\frac{TP+TN}{TP+TN+FN+FP},\\ Recall & =\frac{TP}{TP+FN},\\ Macro\;F1 & =2*\frac{Accuracy*Recall}{Accuracy+Recall}. \end{array}$$

where TP represents the predicted fault-free situation for samples without faults, FP represents the predicted fault-free situation for samples with faults, FN represents the predicted fault-free situation for samples without faults, and TN represents the predicted fault-free situation for samples with faults.

## 4. Simulation Results and Discussion

### 4.1. Feature Selection Results

**Figure 6:**
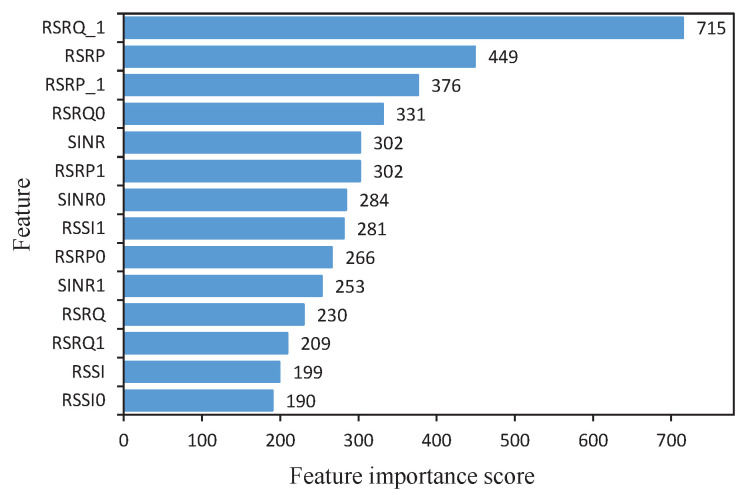
The feature attributes importance scores.

In this paper, the importance score of each feature is obtained through the feature importance ranking function of XGBoost, with which descending ranking is performed. The experimental results are shown in Figure 6.

Next, the optimal subset of network feature parameters needs to be selected based on the importance scores of the network parameters shown in Figure 6. In the feature selection process, XGBoost will continuously increase the feature selection threshold according to the obtained network parameter importance scores and keep the feature parameters whose feature importance score is higher than the threshold; otherwise, it will discard them. In this way, the accuracy of the XGBoost model with different feature combinations is obtained. Finally, the model accuracy and the number of features is judged to obtain the optimal network feature parameter subset. The diagnostic accuracy of the XGBoost model under different numbers of features is shown in Table 3.

It can be seen that the model can obtain better diagnostic accuracy when eight features are selected, and it also achieves the purpose of feature selection. Therefore, in the following simulation experiments of this paper, only the top eight KPI parameters in Figure 6 are set as the KPI parameters.

### 4.2. GAN-Based Data Generation and Data Set Description

In the WGAN-GP model of this paper, the batch size is 50 and the number of iterations is 5000. The specific network parameter settings are shown in Table 4.

**Figure 7:**
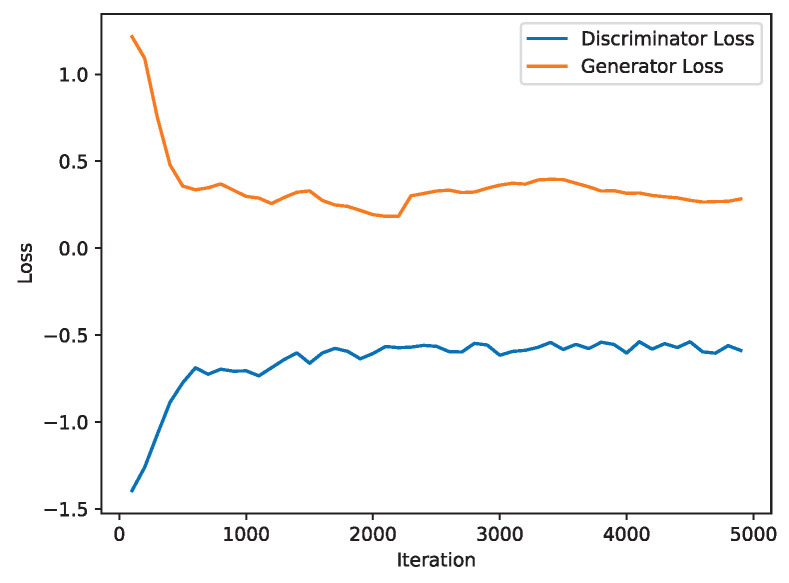
Loss function diagram.

As described in Section 3.2, we generate a large amount of simulated data that match the actual data distribution using WGAN-GP. In the training process of WGAN-GP, the model’s training process can be intuitively reflected by the loss function diagram of the discriminator and the generator. When WGAN-GP is used to fit the actual network data under the network fault scenario of “large station spacing” in the original data set, the loss function of the generator and the discriminator are shown in Figure 7. If the loss value of the discriminator tends to converge, it means that the better the WGAN-GP is trained, the higher the quality of the generated simulated data samples is, and the closer to the distribution of the actual data samples. As seen in Figure 7, the loss function value of the discriminator has a large oscillation at the beginning, then oscillates slightly after it quickly converges. It indicates that the model is in the learning stage at this time, and the model has not yet found the optimal solution direction. After about 2000 training cycles, the model gradually becomes stable, and the gap between generated and real samples gradually decreases. The discriminator gradually cannot distinguish between real and generated samples, and the performance in the graph is gradually becoming stable.

In this paper, we defined eight common faults based on the network optimization rules and fault issue checklist provided by a company’s network optimization personnel. The generated simulated data are merged with the actual data in the original data set to obtain the expanded data set, shown in Table 5. As can be seen from Table 5, the original actual data set is raised to about three times its original size by using WGAN-GP, containing a total of 2657 labeled data. It is noteworthy that when expanding the data for each type of network failure, we try to make the number of samples under each category account for the same proportion of the total number of samples as much as possible so that the samples’ distribution in each category is more uniform. The expanded data set will be applied to the model training process in the subsequent simulation experiments, and the actual data will be selected as the test data set to test the diagnostic accuracy of the model as much as possible.

### 4.3. Continuous KPI Discretization Rules

As mentioned in Section 3.3, since the KPI parameters in the data set obtained through MDT are all continuous-type attributes, if we want to use these data directly for pre-diagnostic classification in the first stage of the naive Bayesian pre-diagnosis process, we need to know the conditional probability density function that the KPI obeys under each network fault state, which is usually challenging to obtain in reality. Hence, the feature parameters need to be further processed, and continuous KPIs are mapped to discrete intervals by reasonably setting thresholds to minimize the missing data information. Thresholds can be defined by experts or learned from training data. Still, in the absence of sufficient data available for training, the former will produce a more accurate model than the latter, so we use expert knowledge to discretize the KPIs. As shown in Table 6, the threshold partition rules are the results of discussions with the company’s network optimization engineers and refer to [4] for some relevant content. In this paper, the KPI feature attributes in the data set are discretized according to the rules in Table 6, after which the likelihood function can be easily calculated by statistical counting based on the frequency of occurrence of the KPI values taken in each network fault state in the training data set, which leads to the final pre-diagnostic classification results in the first stage.

### 4.4. Overall Performance Evaluation and Results

The GCN models in this paper are implemented based on the Keras version, and the simulation experiments are run under the configuration environment of Dell Intel(R) Core(TM) i5-8265U CPU @ 1.60 GHz Round Rock in USA. As described in Section 3.3, the topological association graph between the data obtained based on the pre-diagnosis results has good characteristics and solves the problem of selecting the number of layers of the graph convolution layer in the GCN structure, so the hidden layer depth of all GCN models in the experiments is set to 2. The input feature dimension of the sample data are 8, and the final output feature dimension is 8. The specific GCN network architecture is shown in Table 7. The size of the convolutional filter is selected as 10 and 8, respectively. The learning rate is set to 0.01, the probability of the dropout layer is set to 0.25, and the maximum number of iterations of the neural network training is 200. The L2 regularization parameter is set to $1\times 10^{-5}$. The output of the neural network is completed by propagating the forward excitation to the input. At the same time, the weights $W^{\left(0\right)}$ and $W^{\left(1\right)}$ are updated according to the error back-propagation using batch gradient descent.

**Figure 8:**
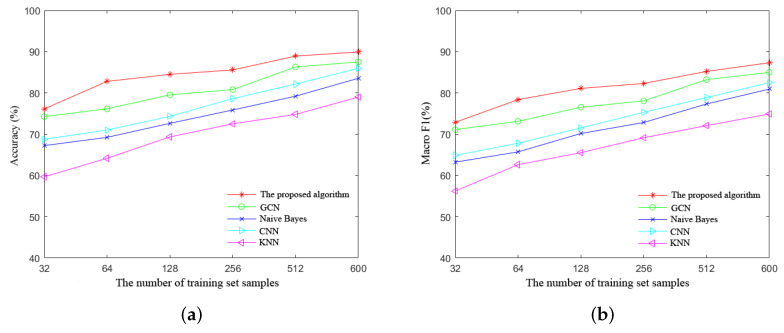
(**a**) Comparison of fault diagnosis accuracy for different algorithms. (**b**) Comparison of fault diagnosis macro F1 for different algorithms.

To demonstrate the effectiveness of the proposed algorithm, we compare it with other algorithms. We compared the parameter settings of CNN [24], KNN [25], and GCN [15] separately. The parameter setting method for naive Bayes has been introduced in Section 4.3. Since the proposed method uses a combination of naive Bayes and graph convolutional neural networks, it is also necessary to compare it with these two algorithms separately. We carry out a total of six groups of experiments; the size of the training set used in each group is 32, 64, 128, 256, 512, and 600, respectively. In the simulation experiments, the model’ s performance will be represented by the accuracy and macro F1 values. Accuracy assesses the overall correctness of a diagnostic model, representing the proportion of correctly classified samples in the predictions. Macro F1, considering the predictive performance for each class and suitable for imbalanced data sets, is the harmonic mean of accuracy and recall. Meanwhile, to reduce the experimental error, the results of each group of experiments are averaged after ten repetitions, and the simulation results are shown in Figure 8a,b.

As can be seen in Figure 8a,b, the proposed algorithm can achieve good accuracy and macro F1 values compared with other algorithms in each group of experiments: the algorithm exhibits a remarkable accuracy of 90.56% and a macro F1 score of 88.41%. It is noteworthy that the proposed algorithm shows better performance compared with either the GCN-based or the naive-Bayes-based network fault diagnosis algorithms alone because the proposed algorithm combines the advantages of the two algorithms and solves their shortcomings in a complementary way.

**Figure 9:**
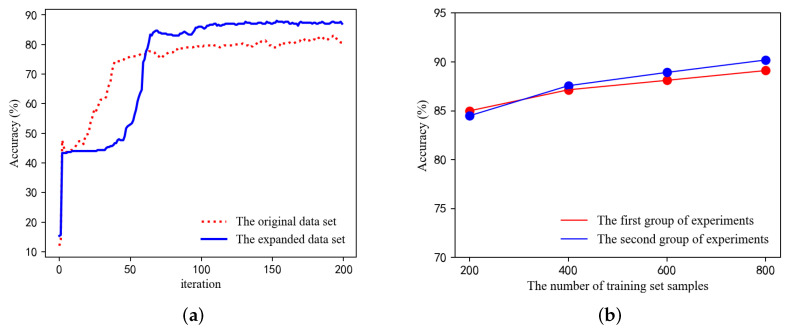
(**a**) The diagnostic accuracy of the proposed algorithm. (**b**) Influence of the weighting coefficient λ on the model.

Next, to verify the impact of the expanded data of the GAN on the accuracy of the overall algorithm model, we conducted experiments based on the original data set and the data set expanded by WGAN-GP to compare the diagnostic accuracy of the proposed algorithm under different situations. The final simulation results are shown in Figure 9a. It can be seen that when using the expanded data set, the simulated data generated by the GAN is indeed consistent with the distribution of actual data in reality, so the algorithm model can obtain sufficient and accurate label information during the training process, which makes the diagnostic accuracy of the model higher.

Finally, we consider the effect of weight coefficient λ on the accuracy of the proposed algorithm. We construct two fault diagnosis models for two groups of experiments, using the same data set expanded by WGAN-GP. The adjacency matrix A used in both sets of experiments is based on the pre-diagnosis results of the first stage of naive Bayes; the only difference is that in the first set of experiments, λ equals 1 (i.e., matrix $\tilde{A}=A+I_{n}$) and then records its accuracy in the training data sets respectively containing 200, 400, 600, and 800 labeled samples. Meanwhile, the forward propagation formula of GCN in the second set of experiments is $H^{\left(l+1\right)}=\sigma \left({\tilde{D}}^{-\frac{1}{2}}\left(A+\lambda I_{n}\right){\tilde{D}}^{-\frac{1}{2}}H^{\left(l\right)}W^{\left(l\right)}\right)$, where $\lambda =1+re^{r}$ is derived from the ratio of the training data set to the total sample data. The results of the experimental simulation are shown in Figure 9b. The results show that the accuracy of the fault diagnosis model using GCN with λ improves slightly with the increase in the number of samples in the training set, which is because the training set has a dominant influence on the accuracy of the model when it is significant. In addition, when the number of labeled samples in the training set is too small, the difference in the accuracy of the model in the two sets of experiments is not significant, which is because the prior knowledge of the pre-diagnosis results has a more significant impact on the accuracy of the model.

## 5. Conclusions

In this paper, we present a knowledge- and data-fusion-based fault diagnosis method for 5G mobile communication networks. To enhance the model’s performance and align it with real-world scenarios, we opt to employ a precise data set containing actual network fault data for the network fault diagnosis task. Given the challenges of sparse labeled samples and uneven distribution in the data set, we leverage GAN to generate synthetic data, thereby expanding the original data set. In the initial phase of the proposed method, to create an accurate topological association graph among the data, we utilize NBM for pre-diagnosing the entire data set and subsequently generate the association graph based on the pre-diagnosis results. In the second phase, the generated association graphs, in conjunction with the enhanced GCN, are employed for the final fault diagnosis of the network fault data set. It is noteworthy that the improved GCN effectively manages and balances the impact of pre-diagnosis results and training data set on the accuracy of the GCN model. The simulation results demonstrate that the algorithm outperforms other traditional models in fault detection and diagnosis tasks, achieving an accuracy of 90.56% and a macro F1 score of 88.41%. Future research will conduct on-site investigations on various network scenarios and expand the types of faults. This can be achieved by collecting more diverse data to adjust and optimize the model and continuously improving the model’s generalizability and robustness.

## Figures and Tables

**Table 1:** Explanations of relevant KPI parameters.

KPI Parameters	Value
RSSI	Received Signal Strength Indicator
RSSI0	the Received Signal Strength Indicator value at channel 0
RSSI1	the Received Signal Strength Indicator value at channel 1
RSRP	Reference Signal Received Power
RSRP0	the Reference Signal Received Power value at channel 0
RSRP1	the Reference Signal Received Power value at channel 1
RSRQ	Reference Signal Received Quality
RSRQ0	the Reference Signal Received Quality value at channel 0
RSRQ1	the Reference Signal Received Quality value at channel 1
SINR	Signal-to-Interference-Plus-Noise Ratio
SINR0	the Signal-to-Interference-Plus-Noise Ratio value at channel 0
SINR1	the Signal-to-Interference-Plus-Noise Ratio value at channel 1
RSRP_1	the maximum Reference Signal Received Power value
RSRQ_1	the maximum Reference Signal Received Quality value

**Table 2:** Network fault types codes.

Category Label	Type of Network Failure	Code
1	signal leakage of the indoor distribution system	1 0 0 0 0 0 0 0
2	measurement threshold abnormality	0 1 0 0 0 0 0 0
3	large station spacing	0 0 1 0 0 0 0 0
4	mode-3 interference	0 0 0 1 0 0 0 0
5	handover threshold abnormality	0 0 0 0 1 0 0 0
6	pilot pollution	0 0 0 0 0 1 0 0
7	overlapping coverage	0 0 0 0 0 0 1 0
8	missing neighbor	0 0 0 0 0 0 0 1

**Table 3:** Diagnostic accuracy of the model with different numbers of features.

Feature Selection Threshold	Number of Features	Accuracy
190	14	86.54%
199	13	86.76%
209	12	86.60%
230	11	86.52%
253	10	86.12%
266	9	86.30%
281	8	86.70%
284	7	85.44%
302	6	84.35%
302	5	83.39%
331	4	81.53%
376	3	81.17%
449	2	79.60%
715	1	77.01%

**Table 4:** WGAN-GP parameter settings.

Model	Number of Layers	Weight Matrix	Offset Vector Length
Generator *G*	1	14 × 32	32
	2	32 × 128	128
	3	128 × 14	14
Discriminator *D*	1	14 × 128	128
	2	128 × 32	32
	3	32 × 1	1

**Table 5:** Data distribution of the data set after expansion with WGAN-GP.

Category Label	Type of Network Failure	Number of Samples
1	signal leakage of the indoor distribution system	347
2	measurement threshold abnormality	342
3	large station spacing	239
4	mode-3 interference	356
5	handover threshold abnormality	300
6	pilot pollution	214
7	overlapping coverage	413
8	missing neighbor	446

**Table 6:** KPI discretization rules.

Discretization Code	RSRP(dBm)	RSRQ(dB)	RSSI(dBm)	SINR(dB)
1	≤−115	≤−20	≤−100	≤3
2	(−115,−105]	(−20,−15]	(−100,−85]	(3,10]
3	(−105,−95]	(−15,−10]	(−85,−70]	(10,15]
4	(−95,−85]	>−10	(−70,−55]	(15,25]
5	>−85	/	>−55	>25

**Table 7:** Graph convolutional neural network architecture.

Number of Layers	Layer Type	Output Feature Size
1	Input layer	2657 × 8
2	Dropout layer 1 (rate = 0.25)	2657 × 8
3	Graph Convolutional Layer 1	2657 × 10
4	Dropout layer 2 (rate = 0.25)	2657 × 10
5	Graph Convolutional Layer 2	2657 × 8
6	SoftMax layer	2657 × 8

## Data Availability

No additional data are available.
